# 152. Sharp Decline in Rates of Community Respiratory Viral Infections Among NIH Clinical Center Patients During the COVID-19 Pandemic

**DOI:** 10.1093/ofid/ofab466.152

**Published:** 2021-12-04

**Authors:** Michele Woolbert, Ninet Sinaii, Christine Spalding, David K Henderson, Tara N Palmore

**Affiliations:** 1 National Institutes of Health, Bethesda, Maryland; 2 NIH, Bethesda, MD; 3 NIH Clinical Center, Maryland

## Abstract

**Background:**

During the first year of the COVID-19 pandemic, nonpharmaceutical interventions had a broad impact on viral transmission apart from SARS-CoV-2. The NIH Clinical Center has used the BioFire FilmArray multiplex PCR respiratory pathogen panel (RPP) for evaluation of upper respiratory symptoms since 2014. Beginning in 3/20, respiratory samples from symptomatic patients were tested by SARS-CoV-2 PCR and the RPP. We performed a retrospective study comparing frequency and rates of community respiratory viruses detected by RPP from 1/14 through 3/21.

**Methods:**

Results of RPPs from nasopharyngeal swabs/washes, bronchoalveolar lavages, and bronchial washes were included. Results from viral challenge studies were excluded. Charts were reviewed to determine whether repeat positives for the same virus within 12 months represented new infections; repeats from the same infection were excluded. A quantitative data analysis was completed using cross tabulations; comparisons were done using mixed models, applying Dunnett’s correction for multiplicity.

**Results:**

A total of 3,329 patients underwent 8,122 RPPs from 1/14 through 3/21. Frequency of all respiratory pathogens declined from an annual range of 0.88-1.97% from 1/14-3/20 to 0.29% in 4/20-3/21 (p < 0.001). Individual viral pathogens declined sharply in frequency during the pandemic, with zero cases of influenza A/B, parainfluenza, or metapneumovirus detected from 4/20-3/21. One case each of adenovirus, RSV, CoV OC43, and CoV HKU1 were detected in 4/20-3/21. Rhino/enterovirus detection continued, but with a substantially lower frequency of 4.27% in 4/20-3/21, compared with an annual range of 8.65-18.28% from 1/14-3/20 (p < 0.001).

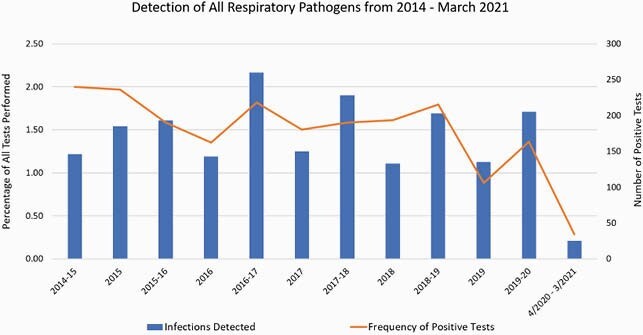

Frequency of detection of all respiratory pathogens tested using the Biofire FilmArray multiplex PCR respiratory pathogen panel from January 2014 through March 2021. The frequency of pathogen detection from April 2020 through March 2021 declined substantially in comparison with previous years.

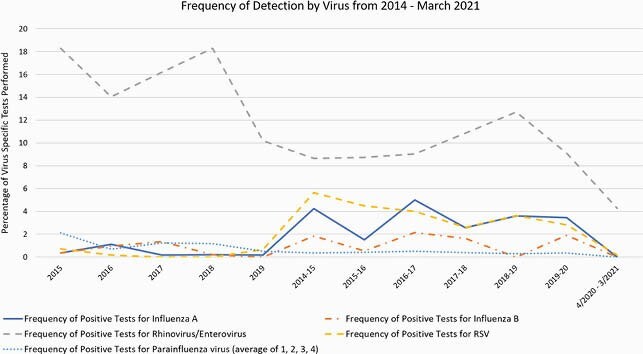

Frequency of detection of influenza A, influenza B, rhinovirus/enterovirus, parainfluenza (1, 2, 3, 4), and respiratory syncytial virus from January 2014 through March 2021. The frequency of detection of these pathogens declined sharply starting in April 2020.

**Conclusion:**

During the pandemic, the burden of viral respiratory infections detected among patients at the NIH Clinical Center improved considerably. This reprieve was likely thanks to the layered COVID-19 prevention and mitigation measures implemented in the community and the hospital: masking, distancing, symptom screening, isolation and testing symptomatic persons. As COVID-19 vaccination allows relaxation of masking, community transmission of respiratory viruses will likely resume; continued mask-wearing in the hospital may provide an enduring benefit by preventing nosocomial transmission.

**Disclosures:**

**Tara N. Palmore, MD**, Nothing to disclose

